# Safety behaviours in social anxiety: an examination across adolescence

**DOI:** 10.1016/j.brat.2021.103931

**Published:** 2021-07-15

**Authors:** Rachel Evans, Kenny Chiu, David M. Clark, Polly Waite, Eleanor Leigh

**Affiliations:** Department of Psychology, Institute of Psychiatry, Psychology & Neuroscience, King’s College London, London, UK; Department of Psychology, Institute of Psychiatry, Psychology & Neuroscience, King’s College London, London, UK; Department of Experimental Psychology, University of Oxford, Oxford, UK; School of Psychology and Clinical Language Sciences, University of Reading, UK; Department of Experimental Psychology, University of Oxford, Oxford, UK; Department of Psychiatry, University of Oxford, Oxford, UK; Department of Experimental Psychology, University of Oxford, Oxford, UK

## Abstract

**Background:**

Safety behaviours have been shown to be a key maintaining factor in Social Anxiety Disorder (SAD). In adults, a two-factor structure of safety behaviours reflecting ‘avoidance’ and ‘impression-management’ types has been identified. This has not yet been investigated in adolescents.

**Aims:**

We set out to investigate the factor structure of safety behaviours in relation to adolescent social anxiety symptoms and SAD, the extent to which this varies by age, and then to examine the association between the derived factor scores and other social anxiety related phenomena.

**Method:**

Questionnaire measures of social anxiety symptoms, cognitions and safety behaviours, peer relationship outcomes, general anxiety and depression were collected from a community sample of 584 younger (11–14 years) and 208 older (16–18 years) adolescents, and a clinical sample of 80 adolescents (11–18 years) with a primary diagnosis of SAD. Four hypotheses were investigated using exploratory and confirmatory factor analyses, regressions, correlations and path analyses.

**Results:**

A two-factor structure reflecting ‘avoidance’ and ‘impression-management’ safety behaviours was supported in the community and clinical sample. Older adolescents were found to use ‘impression-management’ behaviours more than younger adolescents after controlling for overall safety behaviour score. Both types of safety behaviour were significantly positively associated with social anxiety symptoms and cognitions. Path analyses revealed-an indirect effect of social anxiety symptoms on peer victimisation, social satisfaction and friendship quality via ‘avoidance’, but not ‘impression-management’ safety behaviours.

**Conclusions:**

Both ‘avoidance’ and ‘impression-management’ safety behaviours are associated with social anxiety symptoms and cognitions in youth, with age-related differences in their frequency. ‘Avoidance’ behaviours are specifically associated with negative outcomes for quality of peer relationships.

## Introduction

Social anxiety disorder (SAD) is characterised by intense fear of embarrassment or negative judgement by other people, causing distress and functional impairment. It has an average age of onset of 11-13 years (Asher et al., 2017; Kessler et al., 2005) and is very persistent in the absence of treatment (Beesdo-Baum et al., 2012; Bruce et al., 2005; Hale et al., 2008; Wittchen & Fehm, 2003). SAD has been identified as one of the top five functionally impairing psychological disorders (Alonso et al., 2004). For the treatment of SAD in children and adolescents, a well-established and widely used approach is generic forms of cognitive behavioural therapy (CBT), such as ‘The C.A.T. Project’ (Kendall, 2002). These generic treatments were designed to treat common anxiety presentations (e.g. generalised anxiety disorder, separation anxiety disorder, social anxiety disorder) rather than being based on a disorder-specific model. There is growing evidence that youth with SAD experience poorer outcomes from generic CBT compared to youth with other anxiety disorders (Evans et al., in press; Ginsburg et al., 2011; Hudson et al., 2015; Kerns et al., 2013). Identifying modifiable maintenance factors of SAD in youth may hold promise for developing more efficacious interventions for this population.

There is a range of empirically-supported cognitive behavioural models of the maintenance of SAD in adults (Clark & Wells, 1995; Heimberg et al., 2010; Hofmann, 2007; Moscovitch, 2009; Rapee & Heimberg, 1997) which have led to the development of effective psychological treatments (Mayo-Wilson et al., 2014). These models share a number of common factors (Wong & Rapee, 2016). At the heart of all cognitive behavioural models of SAD are social anxiety-related cognitions, which are dysfunctional beleifs and assumptions people have about their social performance and others’ reactions to them in social situations (e.g. “I will blush”, “people will laugh at me”). A number of further factors are thought to maintain these unhelpful cognitions. One such factor common across all of these models is ‘safety behaviours’. Safety behaviours are intended to prevent or mitigate feared outcomes, but in fact prevent people from discovering that their fears are unrealistic and as a consequence inadvertently maintain or worsen anxiety symptoms (Salkovskis, 1991). For example, someone with SAD may censor what they are going to say to other people in order to avoid appearing stupid, but as a consequence they fail to discover that they may have been accepted even if they had said whatever came into their mind and so their anxiety persists. In adults, safety behaviours in SAD have been show to have a range of unintended negative consequences such as increasing state anxiety (Gray et al., 2019); preventing disconfirmation of negative predictions (Wells et al., 1995; McManus et al., 2008); inadvertendly increasing the outward appearance of social anxiety symptoms (Gray et al., 2019; McManus et al., 2008) and negatively affecting the quality of social interactions (Stangier et al., 2006).

Factor analysis of SAD-related safety behaviours in community and clinical samples of adults (using the Social Behaviour Questionnaire; SBQ; Clark, 2005) has shown that these safety behaviours fall into two types (Gray et al., 2019; Plasencia et al., 2011). Some safety behaviours reflect attempts to conceal, hide or limit social engagement, for example, avoidance of eye contact or staying on the edge of social groups (hereafter ‘avoidance’ safety behaviours). Other safety behaviours reflect attempts to create a good social impression, for example rehearsing sentences or closely monitoring one’s behaviour (hereafter ‘impression-management’ safety behaviours). In adults, both ‘avoidance’ and ‘impression-management’ safety behaviours appear to be implicated in the maintenance of SAD. In laboratory conversations, it has been shown that use of both ‘avoidance’ and ‘impression-management’ safety behaviours is associated with greater self-reported anxiety (Gray et al., 2019) and lower self-reported authenticity (Plasencia et al., 2011). However, there is growing evidence in adult samples that these safety behaviours have divergent effects on other people and the quality of social interactions. In laboratory conversations, participants have been rated more critically by conversation partners, including being rated as more anxious and less likeable, when engaging in ‘avoidance’ compared to ‘impression management’ safety behaviours (Hirsch et al., 2004; Gray et al., Plasencia et al., 2011). These findings have key implications for the treatment of SAD. Patients are likely to have experienced negative implicit or explicit feedback as a result of engaging in ‘avoidance’ safety behaviours. This may present a challenge in supporting clients to re-engage in social interactions during therapy.

Although cognitive models of SAD were initially developed in reference to adults, there is evidence supporting the applicability of these models, including the role of safety behaviours, to adolescence (Hodson et al., 2008; Leigh & Clark, 2018; Ranta et al., 2014; Schreiber et al., 2012). As well as being shown to be predictive of social anxiety symptoms, safety behaviours have also been associated with social anxiety-related cognitions (Hodson et al., 2008; Leigh & Clark, 2018; Ranta et al., 2014; Schreiber et al., 2012) which are another common maintenance factor across cognitive behavioural models of SAD (Wong & Rapee, 2016).

The question of whether the two types of safety behaviours identified in adults (‘avoidance’ and ‘impression-management’) manifest similarly in adolescence is yet to be addressed. Given that there is considerable support for the cognitive behavioural processes specified in adult SAD in adolescents (Hodson et al., 2008; Leigh & Clark, 2018; Ranta et al., 2014; Schreiber et al., 2012), it might be expected that the two types of safety behaviours found in adults may also translate to adolescents. However, we must not overlook the range of developmental changes which occur during this stage. During adolescence, neurocognitive changes support the development of key social and cognitive capabilities such as perspective taking, self-awareness, the processing of emotional expressions, emotion regulation, peer influence and sensitivity to social rejection (Blakemore & Choudhury, 2006; Burnett et al., 2011; Haller et al., 2015). It is likely that the development of these social and cognitive capabilities will influence the presentation of safety behaviours (Leigh & Clark, 2018). ‘Impression-management’ safety behaviours such as checking that one is coming across well require cognitive skills such as an understanding of others’ emotional expressions and perspective-taking. In contrast, ‘avoidance’ safety behaviours such as avoiding eye contact are aimed at reducing social contact rather than attempting to act based on an understanding of others’ perceptions and expressions, and as such are unlikely to draw on the same sophisticated level of social and cognitive skills. Therefore, it is possible that whilst both kind of safety behaviour are in operation in adolescence, there are age-related differences in their frequency. Specifically, it may be that impression-management will be observed more frequently in older compared to younger adolescents (Leigh & Clark, 2018).

The present study aimed to establish whether these two types of safety behaviours (‘avoidance’ and ‘impression-management’) occur in adolescence, which the World Health Organisation (WHO) defines as ranging from 10-19 years of age (WHO, 2020) and how they relate to both internal experiences and the quality of peer relationships. Two similar measures of safety behaviours have been developed; the Subtle Avoidance Frequency Examination (SAFE, Cuming et al., 2009) and the Social Behaviour Questionnaire (SBQ; Clark, 2005). To enable comparison with previous studies of the factor structure of safety behaviours in SAD (Gray et al., 2019; Plasencia et al., 2011), we opted to use the Safety Behaviour Questionnaire (SBQ; Clark, 2005). We set out to investigate these issues in a questionnaire-based study with three samples of adolescents; two community samples (11–14 years, N = 584; and 16–18 years, N = 208), and a clinical sample (11–18 years, N = 208). The use of the community samples ensured a sufficient sample size to undertake appropriate analysis to address the research questions, whilst the use of the clinical sample enabled analysis of whether the findings were also observed in adolescents with SAD. Whilst there is good reason to expect that these processes will vary continuously across the population, as demonstrated in adults (Stopa & Clark, 2001), this cannot be assumed and so the inclusion of a clinical sample was important. We investigated the following four hypotheses, with the first two pertaining to community and clinical samples and the second two pertaining to the community samples only due to the clinical data available for secondary analysis:

1. A two-factor model of safety behaviours reflecting ‘avoidance’ and ‘impression-management’ groupings, similar to the structure found in adults will be found in a community and clinical sample of adolescents.
2. In both the community and clinical samples, both ‘avoidance’ and ‘impression-management’ will be significantly positively correlated with social anxiety symptoms and social anxiety-related cognitions.
3. In the community sample, there will be differential associations between the two safety behaviour factors and social outcomes. Specifically, we predict that ‘avoidance’ but not ‘impression-management’ factor scores will be significantly positively associated with self-reported peer victimisation and negatively associated with self-reported friendship quality. We hypothesise that both types of safety behaviour will be negatively associated with social satisfaction because we predict both to be associated with social anxiety symptoms and cognitions.
4. In the community sample, older (16–18 years) adolescents will show relatively greater levels of ‘impression-management’ safety behaviours compared to younger adolescents (11–14 years).

## Method

### Overview and Design

Questionnaire measures of social anxiety symptoms, cognitions and safety behaviours, friendship quality, peer victimisation, social satisfaction as well as depression and anxiety were collected from three samples participating in research projects related to adolescent social anxiety. All participants completed self-report questionnaires including the Social Behaviour Questionnaire (SBQ) at one time point for each participant.

### Recruitment and Sample

#### Community Sample

For the younger community sample, participants were recruited from two mainstream secondary schools. In the two schools, 8.8% and 18.5% of the pupils were eligible for free school meals, an index of social deprivation, which compares to 15.9% on average across schools in England. Participants were 584 young people aged 11–14 years (M = 12.72, SD = 1.98). Participants were 53% female and 45% male. For the older community sample, participants were recruited from a mainstream sixth form college. Participants were 208 young people aged 16–18 years (M = 17.12, SD = .72). Older community sample participants were 57% female and 41% male. Exclusion criteria were i) significant difficulties reading and writing, ii) a diagnosis of autism spectrum disorder (ASD), or iii) a diagnosis of attention deficit hyperactivity disorder (ADHD). For recruitment, classes were identified by schools/colleges based on staff and timetable availability. All students in these classes were invited to hear about the project, consent/assent if they wished to and completed the questionnaires during lesson time.

#### Clinical Sample

Participants were 80 adolescents with a primary diagnosis of SAD as determined by the Anxiety Disorder Interview Schedule (ADIS-IV-C/P; Silverman & Albano, 1996). The clinical sample participants were 84% female and 16% male, and aged 11–18 years (M = 15.26, SD = 1.43). These participants were recruited in clinical research studies by the research group which enabled secondary analysis for the present study.

### Consent and Ethics

#### Community Sample

Ethical approval was granted by University of Oxford Medical Sciences Interdivisional Research Ethics Committee (R54283/RE001; 1^st^ November 2017) and by King’s College London Psychiatry, Nursing and Midwifery Research Ethics Committee on 30^th^ November 2018 (HR-18/19-8278; 30^th^ November 2018). Parental opt-out consent and youth assent methodology was used for 11–14 year old participants and adolescent consent was used for 16–18 year old participants.

#### Clinical Sample

Ethical approval was granted by the NHS Health Research Authority (15/SC/0081; 20^th^ April 2015); University of Reading Research Ethics Committee (15/27; 13^rd^ May 2015;) and the University of Oxford Research Ethics Committee (R60464/RE001; 22^nd^ November 2018). For participants recruited via University of Reading, youth assent (if aged under 16)/consent (if aged over 16) and parental consent was required for participation. For participants recruited via the University of Oxford, parental consent and youth assent was required for participants aged below 16 and youth consent only was required for participants aged 16 and over.

### Materials

#### Social anxiety symptom measures

The self-report version of the Liebowitz Social Anxiety Scale for children and adolescents (LSAS-CA; Masia-Warner et al., 2003) comprises descriptions of 24 social situations and asks respondents to rate their anxiety (on a scale of 0–4) and avoidance (on a scale of 0–4) of each, generating a total score ranging from 0–144. The LSAS-CA was completed by all samples. It had excellent internal consistency in the community sample (α = .97, ω =.97) and clinical sample (α = .96, ω =.96) and has been found to have good divergent and convergent reliability (Storch et al., 2006) as well as good test-retest reliability (Masia-Warner et al., 2003).

#### General anxiety and mood measures

For the community sample, general anxiety was measured using the Screen for Child Anxiety Related Disorders (SCARED; Birmaher et al., 1997) and mood was measured using The Short Mood and Feelings Questionnaire (SMFQ; Angold et al., 1995). For the clinical sample, the Revised Children’s Anxiety and Depression Scale (RCADS-C; Chorpita et al., 2000) was used as a measure of general anxiety and mood. The SCARED (Birmaher et al., 1997) comprises 41 items measuring anxiety on a scale of 0-2. It is a widely used measure with good psychometric properties (Birmaher et al., 1997). To enable analyses that controlled for anxiety symptoms not due to social concerns (hereafter referred to as ‘general anxiety’) we opted to calculate a total SCARED score excluding the 7 social anxiety items. This yielded a total SCARED General Anxiety score of 0–68. The SMFQ (Angold et al., 1995) comprises 13 items related to low mood on a scale of 0–2, with a total score of 0–26. The SMFQ is also widely used and has been shown to have strong psychometric properties (Thabrew et al., 2018). The RCADS-C (Chorpita et al., 2000) is a widely used 47-item scale comprising five anxiety subscales and depression scale, with good psychometric properties (Kösters et al., 2015). For reasons outlined above, to generate a score of general anxiety (anxiety not due to social concerns) we calculated an RCADS General Anxiety score by summing the anxiety subscales excluding the 9 social anxiety items, yielding a total RCADS General Anxiety score of 0–84. The RCADS Depression scale consists of 10 items yielding a total RCADS Depression scale score of 0–30. Community samples completed the SCARED (α = .95, ω =.96) and SMFQ (α = .96, ω =.92) and the clinical sample completed the RCADS-C (RCADS-C Total α = .94, ω =.95; RCADS-C General Anxiety α = .92, ω =.93; RCADS-C Low Mood Scale α = .85, ω =.85).

#### Diagnostic measure

The Anxiety Disorders Interview Schedule – Child and Parent Versions (ADIS-IV-C/P; Silverman & Albano, 1996) is a semi-structured interview used to assess for anxiety disorder diagnoses, on the basis of both child and parent report, and is widely used to assess outcomes in treatment trials for child and adolescent anxiety (Ginsburg et al., 2011; Hudson et al., 2014). The ADIS-IV-C/P has been shown to have good test-retest reliability and concurrent validity (Silverman et al., 2001; Wood et al., 2002). The ADIS-IV-C/P was completed by the clinical sample only.

#### Safety behaviour measure

The Social Behaviour Questionnaire (SBQ; Clark, 2005) is a 28-item scale measuring use of a range of safety behaviours in social situations and asks respondents to report frequency of use on a scale of 0 (‘not at all’) – 3 (‘very frequent’). An overall SBQ score is calculated as a mean of responses, with a range of 0 (no safety behaviours) to 3 (very frequent safety behaviours). The SBQ was completed by the community sample (α = .91, ω =.90) and clinical sample (α = .85, ω =.85).

#### Social cognitions measure

The Child & Adolescent Social Cognitions Questionnaire (CASCQ; Leigh & Clark, 2021; adapted from the adult SCQ; Clark, 2005) contains 29 social anxiety-related cognitions. The CASCQ is highly similar to the adult SCQ with the addition of some items considered to be relevant to youth (e.g. “people won’t want to be friends with me”) and changes to make the wording more accessible (e.g. “I am inadequate” becomes “I am not good enough”). Respondents rate the frequency of these cognitions on a scale of 1–5 and their belief in them on a scale of 0-100. In the present study, the CASCQ mean belief rating was selected as a measure of social anxiety-related cognitions, as strength of cognition may be a more clinically meaningful index of social beliefs. The CASCQ was completed by the community sample (α = .98, ω =.97), and clinical sample (α = .84, ω =.87).

#### Measures of social outcomes

The Friendship Quality Questionnaire (FQQ; Parker & Asher, 1993) contains 40 statements about friendships and asks respondents to rate each on a scale of 0–4, with higher scores indicating better friendship quality. The Validation and Caring subscale of the FQQ (10 items; range: 0–40) was selected for use in the study. This is because items in this subscale were considered to show the least overlap with ‘impression management’ safety behaviours. Items in the other five subscales of the FQQ refer to the participant’s own behaviour or dyadic behaviour (e.g. “Loan each other things all the time”) and so arguably overlap with ‘impression management’ safety behaviours, whereas items in the Validation & Caring subscale assess how the specific friend relates to the participant (e.g. “Makes me feel good about my ideas”, “Cares about my feelings”). The FQQ has been shown to be a reliable measure of friendship quality in youth (Aikins et al., 2005). The Social Satisfaction Scale lists a range of typical social relationships and asks respondents to rate their satisfaction with them on a scale of 1-7, with higher scores indicating greater social satisfaction. The original Social Satisfaction Scale contains five items including one on romantic relationships, however given the age of the current sample this item was dropped, and a four-item version of the Social Satisfaction Scale was used (range 4-28). The Peer Victimisation Measure (Tillfors et al., 2012) comprises three items regarding mocking, assault and social exclusion. Responses are rated on a scale of 1–4, generating a total score of 3–12. These four measures of social outcomes were collected from the community sample only (FQQ Validation & Caring Subscale, α = .88, ω =.88; Social Satisfaction Scale, α =.79, ω =.79; Peer Victimisation Measure, α = .59, ω =.64). The relatively low internal consistency for the Peer Victimisation Measures is likely due to it being only a 3-item measure, with Cronbach’s Alpha particularly sensitive to the number of scale items (Field, 2009).

### Data Analysis Plan

Data were analysed using SPSS version 26 (IBM Corp, 2019) and R version 3.6.2 (R Core Team, 2019), with R packages of ‘Psych’ (Revelle, 2019), ‘Lavaan’ (Rosseel, 2012) and ‘MASS’ (Ripley et al., 2013). Prior to all analyses, data were checked for the assumptions of the relevant statistical tests. In cases where the assumption of normality was violated, non-parametric tests were used.

To examine the factor structure of the Safety Behaviour Questionnaire (Hypothesis 1), the community sample data were randomly split into approximate halves to enable Exploratory Factor Analysis (EFA) on the first half (N=409) and Confirmatory Factor Analysis (CFA) on the second half (N=383) of the data set. The Kaiser-Meyer-Olkin (KMO) measure of sampling adequacy (KMO = .92) and Bartlett test of sphericity (*χ* ^2^ (378) = 4019.48, *p* < .001) were examined to confirm that the data were suitable for EFA. A cut-off factor loading of .4 was pre-determined (Gray et al., 2019; Plasencia et al., 2011; Laher, 2010). Parallel analysis and the scree plot were consulted to determine the number of factors to be extracted. As the factors were anticipated to be correlated (Gray et al., 2019), oblique (Direct Oblimin) rotation was used. As the SBQ provides ordinal data, the weighted least square mean and variance (WLSMV) method was used (Li, 2016). The fit of the factor structure established from EFA was then assessed using CFA in the second half (N=383) of the community sample. It was pre-determined that the fit would be assessed against the robust Comparative Fit Index (CFI), Tucker-Lewis Index (TLI), Root Mean Square Error of Approximation (RMSEA) and the Standardised Root Mean Squared Residual against recommended cut-offs (Hu and Bentler, 1999; Schreiber et al., 2006) of close to .95 for TLI and CFI, .06–.08 for RMSEA and close to .08 for SRMR. CFA was then repeated in the whole community sample to derive factor scores for each participant. The CFA was repeated in the clinical sample to establish the fit of the factor structure in the clinical sample and attain factor scores for each participant in this sample.

To examine the association between ‘avoidance’, ‘impression-management’, social anxiety symptoms and cognitions (Hypothesis 2), we completed partial Spearman’s Rho correlations investigating the association between social anxiety symptoms and factor scores whilst controlling for depression and general anxiety, and the association between factor scores and social anxiety-related cognitions whilst controlling for social anxiety symptoms. To examine the association between ‘avoidance’, ‘impression-management’ and measures of social relationships (Hypothesis 3) path analysis was undertaken with the ‘lavaan’ package in R (Rosseel, 2012). Bootstrapping with 1000 replications was applied to derive more accurate standard errors and bias-corrected confidence intervals were computed. Variables were standardised prior to analysis. As a sensitivity analysis to account for the nested nature of community sample data within schools, path analyses were also rerun with school as a categorical variable (McNeish & Stapleton, 2016). School was not a significant covariate in any of the analyses (p>.05) nor did it change any of the findings, therefore results are presented without this taken into account. To examine age-related differences in safety behaviour frequency (Hypothesis 4), multiple linear regressions were used to examine the association between factor scores and age group. Diagnostic plots and variance inflation factors were consulted to confirm that the assumptions of homoscedasticity, normal distribution of residuals, linearity and multicollinearity were met for the regression models.

### Missing Data

For the calculation of questionnaire total scores, mean replacement was used for missing data in cases where at least 80% of questionnaire responses were complete. Average missing data at the variable level (questionnaire totals) was low across the samples (Younger Community = 3%, Older Community = 1%, Clinical = 2%). Missing data at the item level during factor analysis on the SBQ was managed using pairwise deletion. The mean proportion of missing data across the 28 SBQ items was also very low (Younger Community Sample = 0.91%, Older Community Sample = 0.38%, Clinical Sample = 0.54%). For the path analysis to investigate Hypothesis 3, missing data were handled using full information maximum likelihood (FIML) estimation, which adjusts the likelihood function so that each case contributes information on the variables that are observed.

## Results

### Descriptive Statistics

The mean questionnaire scores for all samples are shown in Table 1. Social anxiety symptoms, cognitions and social safety behaviours all increased with age and were greater in the clinical sample compared to the community samples. Shapiro-Wilk tests showed that all questionnaire variables in the community sample, and the LSAS-CA and CASCQ in the clinical sample, were significantly non-normal in distribution (*p* < .05). Comparisons shown between younger and older community sample in Table 1 were therefore calculated using Wilcoxon Rank Sum tests, and comparisons between the three samples were calculated using Kruskal-Wallis tests. Consistent with previous findings (Angold, Costello, & Worthman, 1998; Beesdo, Knappe, & Pine, 2009) older adolescents reported greater scores on measures of general anxiety and depression. Questionnaires used to measure general anxiety and depression in the clinical sample (RCADS General Anxiety M = 33.88, SD = 13.83, RCADS Mood M = 14.58, SD = 5.74) were different to those in the community sample and so are not included in Table 1.

### Hypothesis 1: A two-factor model reflecting ‘avoidance’ and ‘impression-management’ safety behaviours will be supported in the community and clinical samples

#### Exploratory factor analysis

**Figure 1:**
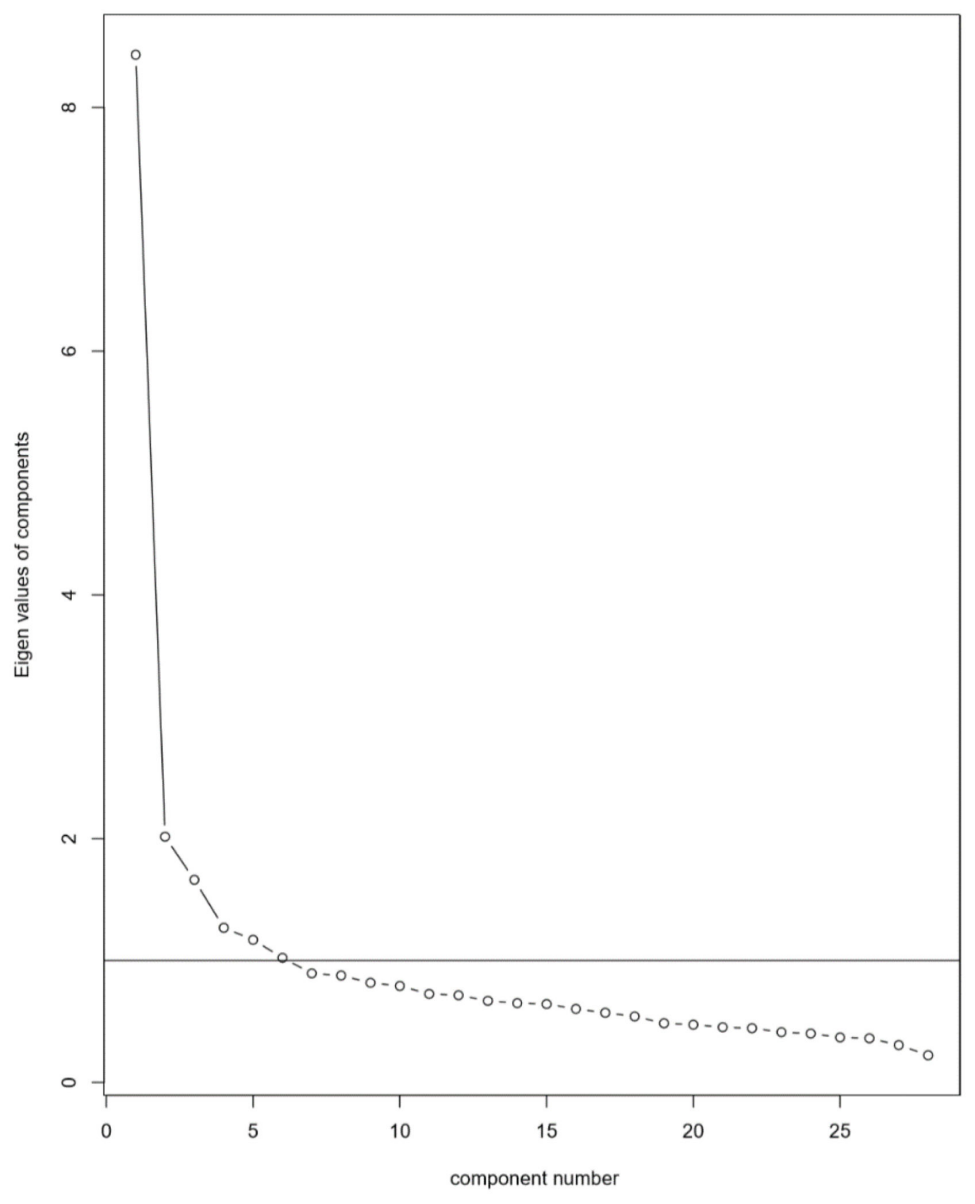
Scree plot showing eigenvalues of extracted components from SBQ data.

Parallel analysis suggested that five factors should be extracted, and the scree plot (Figure 1) suggested that either two or four factors should be extracted. Item loadings were examined for a two, three, four and five factor solution. In the three, four and five factor solutions, the additional variance explained beyond a two-factor solution was minimal (2–6%). The two-factor solution was the most clearly interpretable. Together, these two factors explained 29.3% of the variance (Factor 1 = 18.4%, Factor 2 = 10.8%). For factor loadings, see Table 2. The first of these two factors can be understood to reflect ‘avoidance’ safety behaviours (for example, ‘avoid eye contact’, ‘try not to attract attention’) and the second can be understood to reflect ‘impression-management’ safety behaviours (for example, ‘try to fit in and act normal’, ‘check what you are going to say’). Table 2 also shows the six items which did not load sufficiently onto either factor (marked with an asterisk).

#### Confirmatory Factor Analysis

Confirmatory factor analysis was then completed in the second randomly-selected half of the community sample (N=383). The CFI and TLI for this model in the second half of the sample were .93 and .92, respectively, close to .95 as recommended by Hu and Bentler (1999). The robust root mean square error of approximation (RMSEA) was .08 (95% CI: .07–.08), and the root mean squared residual (SRMR) was .08; indicating an acceptable fit (Hu & Bentler, 1999; Schreiber et al., 2006). All items loaded onto their respective factors with a loading magnitude of > .5, with the exception of one item (“Talk More”) which revealed a very low loading at this stage (.04) and was therefore dropped. Internal consistency was good for both avoidance (α = .87, ω =.87) and impression management (with “Talk More” included α=.77, ω =.79, with “Talk More” excluded α =.79, ω =.81). Confirmatory factor analysis was then repeated in the whole community sample to generate factor scores for each participant (factor loadings are presented in Table 3).

#### Confirmatory Factor Analysis in the Clinical Sample

CFA was used to examine the fit of the two-factor structure in the clinical sample. These results should be understood to be exploratory, as smaller sample size can affect fit indices in CFA (DiStefano, 2002; Marsh, Balla, & McDonald, 1988). The CFI (.91) and TLI (.92) were close to .95 as recommended by Hu and Bentler (1999) and the RMSEA was within the acceptable range (.07) although the upper end of the confidence interval was slightly beyond this range (95% CI:.04–.09). The SRMR was .12, which is above the recommended level of < .08 (Schreiber et al., 2006), which may reflect SRMR being particularly sensitive to small sample size (DiStefano, 2002). Internal consistency was good both for ‘avoidance’ (α =.80, ω =.80) and ‘impression-management’ (α =.82, ω =.81) in the clinical sample. As shown in Table 3, 19/21 items loaded above the .4 cut-off onto the two factors, with an average loading of .59 onto the ‘avoidance’ factor and .69 on the ‘impression-management’ factor. One of the two items (“avoid eye contact”) which did not meet the .4 cut-off was only marginally lower than this threshold at .37, whereas the other (“try to think about other things”) loaded at .16.

### Hypothesis 2: ‘Avoidance’ and ‘Impression-Management’ safety behaviours will be associated with social anxiety symptoms and cognitions

To investigate Hypothesis 2, a series of Spearman’s Rho correlation and partial Spearman’s Rho correlations were conducted between ‘avoidance’, ‘impression-management’, social anxiety symptom severity (LSAS-CA), and social anxiety-related cognitions (CASCQ) in both the community and clinical sample.

#### Community Sample

As shown in Table 4, in the community sample both ‘avoidance’ and ‘impression-management’ were significantly positively correlated with social anxiety symptoms and cognitions as well as anxiety and depression. Partial Spearman’s Rho correlations revealed that after controlling for depression and general anxiety, there remained a significant positive correlation between social anxiety symptoms and both ‘avoidance’, *r*_s_ = .50, *p* <.001, and ‘impression-management’, *r*_s_ = .28, *p* <.001.

As shown in Table 4, both ‘avoidance’ and ‘impression-management’ were also significantly positively correlated with social anxiety-related cognitions. To test the possibility that the association could be explained by the overlap of these constructs with social anxiety symptoms, partial Spearman’s Rho correlation was conducted controlling for social anxiety symptoms. A significant positive correlation was found between social anxiety cognitions and both ‘avoidance’ (*r*_s_ =.38, *p*<.001) and ‘impression-management’ (rs =.34, *p* <.001) after controlling for social anxiety symptoms. Additional analyses revealed that the correlations shown in Table 4 between ‘avoidance’, ‘impression-management’, social anxiety-related cognitions and social anxiety symptoms were in the moderate-strong range in both the younger (*r*_s_ =.59–.73, all *p* <.001) and older community sample (*r*_s_ =.62–.77, all *p* <.001).

#### Clinical Sample

As shown in Table 4, in the clinical sample both ‘avoidance’ and ‘impression-management’ were significantly positively correlated with social anxiety symptoms (LSAS-CA), social anxiety-related cognitions (CASCQ), general anxiety (RCADS-C General Anxiety) and depression symptoms (RCADS-C Depression), with correlations mostly in the moderate to strong range. Partial Spearman’s Rho correlations revealed that after controlling for depression symptoms and general anxiety, there remained a significant positive correlation between social anxiety symptoms and both ‘avoidance’, *r*_s_ = .58, *p* <.001, and ‘impression-management’, *r*_s_ =.28, *p* <.05.

As shown in Table 4, in the clinical sample both ‘avoidance’ and ‘impression-management’ were also significantly positively correlated with social anxiety-related cognitions. Partial Spearman’s Rho correlations were conducted between factor scores and social anxiety-related cognitions in the clinical sample, controlling for social anxiety symptoms. This revealed that social anxiety-related cognitions were significantly positively correlated with both ‘avoidance’ (*r*_s_ = .35, *p* <.05) and ‘impression-management’ (*r*_s_ = .36, *p* <.05), after controlling for social anxiety symptom severity in the clinical sample.

### Hypothesis 3: ‘Avoidance’ and ‘impression-management’ will have diverging associations with measures of social relationship quality

#### Peer Victimisation

**Figure 2:**
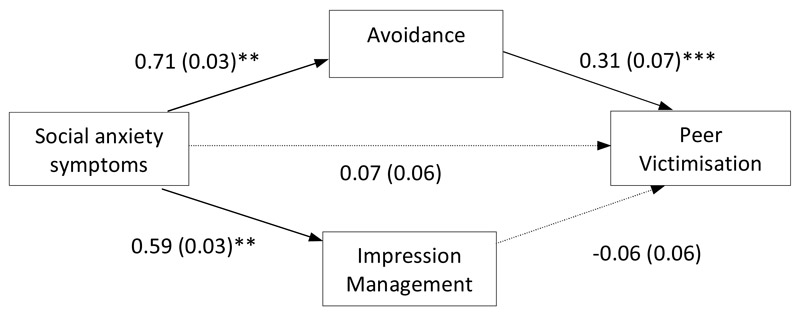
Path analysis model testing the direct effect of social anxiety on peer victimisation and the indirect effect via ‘avoidance’ and impression management’ safety behaviours (age and gender were included as covariates but are not shown here for ease of interpretation). *Note:* Values presented are standardised path coefficients, values presented in parentheses are standard errors. * *p*<.05. ** *p*<.01; *** *p*<.001

The first parallel path analysis model examined social anxiety symptoms as a predictor of peer victimisation, with indirect effects via ‘avoidance’ and ‘impression management’ safety behaviours. The two safety behaviour variables were allowed to correlate. Age and gender were included as covariates. In first-order correlations, there was a significant positive association between social anxiety symptoms and peer victimisation (*r_s_* = .20, *p* < .005). As can be seen in Figure 2, there was an indirect association between social anxiety symptoms and peer victimisation via ‘avoidance’ safety behaviours (b = 0.22 [95% CI: 0.13, 0.32], *SE* = 0.05, *p* < .001), with higher social anxiety symptoms associated with more ‘avoidance’ safety behaviours and in turn, more peer victimisation. In comparison, ‘impression management’ safety behaviours did not have a significant influence on this association (*b* = -0.04 [95% CI: -0.11, 0.03], *SE* = 0.04, *p* > .05).

Given the intercorrelations amongst the three measures of social relationship quality, we reran the path analysis examining peer victimisation, whilst controlling for friendship quality and social satisfaction. Results were unchanged, and the indirect association between social anxiety symptoms and peer victimisation via avoidant safety behaviours remained significant (*b* = 0.15 [95% CI: 0.05, 0.23], *SE* = 0.05, *p* < .001).

#### Social Satisfaction

**Figure 3:**
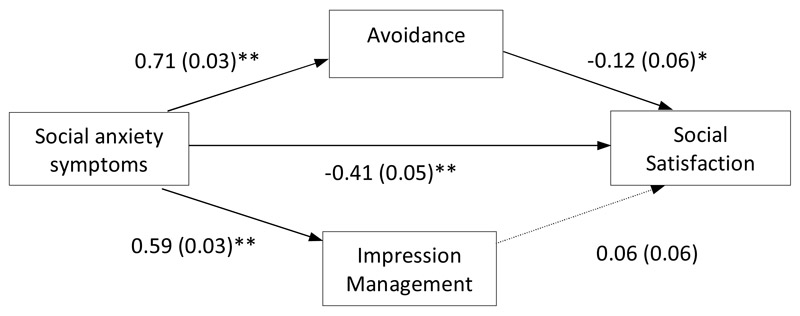
Path analysis model testing the direct effect of social anxiety on social satisfaction and the indirect effect via ‘avoidance’ and ‘impression management’ safety behaviours (age and gender were included as covariates but are not shown here for ease of interpretation). *Note.* Values presented are standardised path coefficients, values presented in parentheses are standard errors. * *p*<.05. ** *p*<.01; *** *p*<.001

The second parallel path analysis model examined social anxiety symptoms as a predictor of social satisfaction, with indirect effects via ‘avoidance’ and ‘impression management’ safety behaviours. The two safety behaviour variables were allowed to correlate. Age and gender were included as covariates. There was a significant negative first-order correlation between social anxiety symptoms and social satisfaction (*r_s_* = -.48, *p* < .005). As can be seen in Figure 3, there was an indirect association between social anxiety symptoms and social satisfaction via ‘avoidance’ safety behaviours (b = -0.09 [95% CI: -0.17, -0.001], *SE* = 0.04, *p* < .05), with higher social anxiety symptoms associated with more ‘avoidance’ safety behaviours and in turn lower selfreported social satisfaction. In contrast, there was no significant influence of ‘impression management’ safety behaviours on the association between social anxiety symptoms and social satisfaction (b = 0.03 [95% CI: -0.03, 0.10], *SE* = 0.03, *p* > .05).

Given the intercorrelations amongst the three measures of social relationship quality, we reran the path analysis examining social satisfaction, whilst controlling for friendship quality and peer victimisation. The indirect association between social anxiety symptoms and social satisfaction via avoidant safety behaviours was no longer significant (*b* = -0.01 [95% CI: -0.09, 0.07], *SE* = 0.04, *p* > .05).

#### Friendship Quality

**Figure 4:**
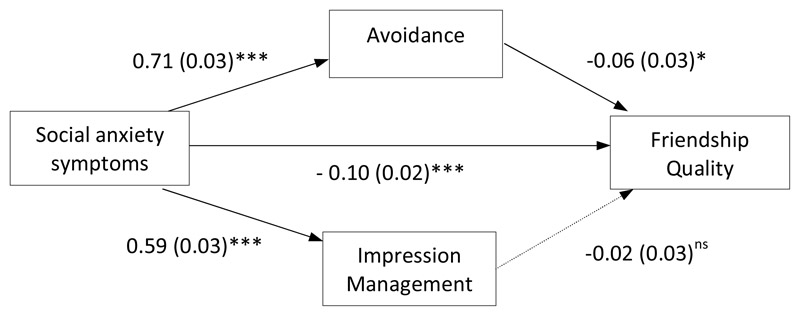
Path analysis model testing the direct effect of social anxiety on the Friendship Quality Questionnaire (Validation & Caring Subscale) and the indirect effect via ‘avoidance’ and ‘impression management’ safety behaviours (age and gender were included as covariates but are not shown here for ease of interpretation). *Notes*: Values presented are standardised path coefficients, values presented in parentheses are standard errors. ^ns^*p* > .05; * *p* < .05; ** *p* < .01; *** *p* < .001

The third parallel path analysis model examined social anxiety symptoms as a predictor of self-reported friendship quality, with indirect effects via ‘avoidance’ and ‘impression management’ safety behaviours. The two safety behaviour variables were allowed to correlate. Age and gender were included as covariates. There was a significant negative first-order correlation between social anxiety symptoms and friendship quality (*r_s_* = -26, *p* < .001). As can be seen in Figure 4, there was an indirect association between social anxiety symptoms and friendship quality via ‘avoidance’ safety behaviours (*b* = -0.04 [95% CI: -0.09, -0.01], *SE* = 0.02, *p* < .05), with higher social anxiety symptoms associated with more ‘avoidance’ safety behaviours, which in turn was associated with poorer friendship quality. ‘Impression management’ safety behaviours did not have a significant influence on the association between social anxiety symptoms and friendship quality (*b* = -0.01 [95% CI: -0.04, 0.02], *SE* = 0.02, *p* > .05).

Given the intercorrelations amongst the three measures of social relationship quality, we reran the path analysis examining friendship quality, whilst controlling for social satisfaction and peer victimisation. Results were unchanged, and the indirect association between social anxiety symptoms and peer victimisation via avoidant safety behaviours remained significant (*b* = -0.18 [95% CI: -0.27, -0.10], *SE* = 0.04, *p* < .001).

### Hypothesis 4: Older adolescents will report relatively greater frequency of ‘impression-management’ safety behaviours compared to younger adolescents

In the community sample, because older adolescents reported greater overall SBQ scores, in order to examine the association between age group factor scores for Hypothesis 4 it was necessary to assess the relationship between age group and both ‘avoidance’ and ‘impression-management’ whilst controlling for average SBQ score. Two multiple linear regressions were conducted to examine age group (younger vs. older) and average SBQ as predictors of ‘avoidance’ and ‘impression-management’. As shown in Table 5, after controlling for overall SBQ score, age group was a significant predictor of ‘impression-management’ but not ‘avoidance’ factor score, with older adolescents using ‘impression-management’ safety behaviours more than younger adolescents.

## Discussion

This study set out to investigate the factor structure of safety behaviours in relation to adolescent social anxiety symptoms and disorder, and the relationship between derived factor scores and social anxiety symptoms and cognitions, peer relationship outcomes, and age group. This revealed that the two-factor structure of ‘avoidance’ and ‘impression-management’ safety behaviours that is evident in adults also applies to adolescents, both in a community sample and a clinical sample. Use of ‘impression-management’ safety behaviours was more prominent in older than younger adolescents. Both types of safety behaviour were strongly related to measures of social anxiety symptoms and social anxiety-related cognitions. However, they diverged in their associations with measures of peer relationship quality.

In support of Hypothesis 1, results from exploratory and confirmatory factor analysis supported a two-factor structure of safety behaviours reflecting ‘avoidance’ (e.g. “avoid asking questions”, “hide your face”) and ‘impression-management’ (e.g. “try to fit in and act normal”, “rehearse sentences in your mind”) categories in the adolescent community sample. Although underpowered, results in the smaller clinical sample also provided an indication that the two-factor structure may be applicable in this population. The two-factor structure of ‘avoidance’ and ‘impression-management’ safety behaviours is consistent with the structure identified in adult samples (Gray et al., 2019; Hirsch et al., 2004; Plasencia et al., 2011). The proportion of variance explained by these two factors in our sample (29.3%) falls in between the 25.79% and 44.55% of variance explained in similar studies in adults conducted by Gray et al. (2019) and Plasencia et al. (2011), respectively. This suggests that there may be other safety behaviour factors beyond ‘avoidance’ and ‘impression-management’ in adolescents, which are yet to be identified.

In support of Hypothesis 2, there were significant positive correlations between ‘avoidance’, ‘impression-management’, social anxiety symptoms and cognitions in the community and clinical sample. Moreover, both ‘avoidance’ and ‘impression-management’ were significantly positively correlated with social anxiety symptoms in both samples after controlling for general anxiety and depression. This suggests that the positive association between factor scores and social anxiety symptoms is not an artefact of their association with general anxiety and depression. Additionally, evidence in the community sample showed that the associations between these variables remains significant with similar magnitude in both the younger and older age groups. These findings complement findings of an association between these types of safety behaviours and internal experiences associated with social anxiety such as low self-authenticity and increased state anxiety in adults (Gray et al., 2019; Plasencia et al., 2011). This is also consistent with previous findings associating safety behaviours with severity of social anxiety symptoms and cognitions in adolescents (Hodson et al., 2008; Ranta et al., 2014; Schreiber et al., 2012). Moreover, by demonstrating that both ‘avoidance’ and ‘impression-management’ safety behaviours are associated with social anxiety-related cognitions, and indeed with social anxiety symptom severity, our results suggest that both of these types of safety behaviours can be understood to fit within the Clark and Wells (1995) maintenance model of SAD as applied to adolescents.

In support of Hypothesis 3, there was a significant indirect positive association between social anxiety symptoms and peer victimisation via ‘avoidance’ but not ‘impression-management’ safety behaviours. There was also a significant indirect negative association between social anxiety symptoms and friendship quality via ‘avoidance’ but not ‘impression-management’ safety behaviours. An unexpected finding in relation to Hypothesis 3 was that ‘avoidance’ but not ‘impression-management’ safety behaviours also had a significant indirect effect on the negative association between social anxiety symptoms and social satisfaction. However, there was no significant indirect association between social anxiety symptoms and social satisfaction via either type of safety behaviour when controlling for the other two peer outcome measures. This contrasts with our prediction that both types of safety behaviours would be associated with poorer social satisfaction. The finding suggests that of both types of safety behaviours, the effect on social satisfaction does not operate in this way. Overall, these findings suggest a clear pattern that ‘avoidance’ but not ‘impression-management’ safety behaviours, in an unselected sample at least, are associated with negative outcomes in terms of their influence on the quality of social interactions and peer relationships.

These results complement previous findings of an association between social anxiety, friendship difficulties and peer victimisation in adolescence based on both self- (Baker & Hudson, 2015; Bernstein et al., 2008) and peer- (Erath et al., 2007; Flanagan et al., 2008) report. To understand this relationship, it is helpful to consider findings from adult research. Several studies in adults (Hirsch et al., 2004; Gray et al., 2019; Plasencia et al., 2011) have shown that in experimental conversations, participants displaying ‘avoidance’ safety behaviours were rated as more anxious and less likeable than partners compared to when not displaying these behaviours. In contrast, this effect was not observed for ‘impression-management’ behaviours. Additionally, in a study of adolescents which examined imagery associated with SAD, Leigh et al. (2020) found that ‘avoidance’ but not ‘impression-management’ safety behaviours (categorised based on Hirsch et al., 2004) mediated the relationship between negative imagery and partner ratings of conversation quality in a laboratory conversation. It is possible that similar peer reactions to ‘avoidance’ behaviours explain our finding regarding peer relationship outcomes. Whilst difficulties with social relationships and victimisation would be distressing for people of any age, these findings have particular significance for an adolescent population. Adolescence is the period in ones’ life in which public selfconsciousness develops and tends to peak (Rankin et al., 2004). At the same time, young people experience heightened sensitivity to peer feedback (Foulkes & Blakemore, 2016; Kilford et al., 2016) and distress in response to rejection (Platt et al., 2013). Therefore, the friendship difficulties and peer victimisation that we found to be associated with ‘avoidance’ safety behaviours are likely to be particularly distressing for this age group.

In support of Hypothesis 4, older adolescents were found to report greater relative use of ‘impression-management’ safety behaviours. Indeed, consistent with previous research (Rao et al., 2007; Westenberg et al., 2007) older adolescents were also found to have significantly higher scores on measures of social anxiety symptoms, as well as social anxiety-related cognitions, depression symptoms and general anxiety symptoms. This provides important evidence of age-related effects on safety behaviours during adolescence and highlights the need to consider developmental changes within adolescence rather than simply comparing findings from adults with those in young people as a homogenous group. A possible explanation for this is that developmental neurocognitive changes which occur during adolescence enable development of skills such as perspective taking and understanding emotional expressions which are required to engage in the relatively sophisticated ‘impression-management’ behaviours such as ‘check that you are coming across well’ and ‘try to fit in and act normal’. In contrast, ‘avoidance’ behaviours such as ‘talk less’ and ‘hide your face’ seem unlikely to require these complex social cognitive skills which develop during adolescence.

This study has a number of strengths. It benefited from a large community sample, which enabled a combination of exploratory and confirmatory factor analysis. It also benefited from the inclusion of a clinical sample. Additionally, participants completed a range of validated and widely used questionnaires. Several limitations also require consideration. The use of self-report questionnaires means that the findings could have been affected by common method variance. In particular, we note that findings of peer functioning based on self-report should be interpreted with caution as they reflect perceptions of rather than objective peer functioning. Future research would therefore benefit from more objective or observer-report measures. The cross-sectional design also limits conclusions on the temporal relationship between safety behaviours and social outcomes, suggesting that future research would benefit from the use of longitudinal designs. Additionally, we were only able to investigate developmental differences using the proxy measure of chronological age, and future research would benefit from the inclusion of specific measures of developmental stage such as measures of pubertal status. We were also not able to report data on the ethnicity or socio-economic status of out sample, which limits conclusions on the generalisability of the results. Furthermore, although the fit indices were generally within the recommended range, some fit indices were marginally outside of recommended cut-offs. This suggests that the factor structure identified in the present study may not provide the most optimal explanation for safety behaviours in adolescent SAD and may be improved in further research. Additionally, the clinical sample was underpowered to fully examine the factor structure in this population. The available data for the clinical sample also did not enable us to investigate any relationship between comorbidity and safety behaviour scores, and it would be beneficial for future research to consider this issue. Finally, the clinical sample was predominantly female, whereas the community sample had a more balanced gender split. Although direct comparisons between the community and clinical samples were not intended, the high proportion of females in the clinical sample should be noted when considering the generalisability of results.

Our results highlight a range of other implications for future research. It would be beneficial to repeat our study with a larger and sufficiently powered clinical sample. Given the finding that these two factors explained 29.3% of the variance in SBQ score, and six items did not load onto either factor, future research should work to examine if other categories of safety behaviours exist beyond ‘avoidance’ and ‘impression-management’ and to consider whether the six items not included in these two factors might be included in other factors. This future research may benefit from also including items from the SAFE questionnaire (Cuming et al., 2009), as this includes some items which are not captured in the SBQ (Clark, 2005) and vice versa. For example, the SAFE includes several items related to making excuses for feared symptoms and this could form a distinct factor alongside impression-management and avoidance. This study’s findings, alongside those of Leigh et al. (2020) regarding an association between mental imagery and social anxiety in adolescents, suggest further examination of the associations amongst these processes (Hirsch, Clark & Matthews, 2006) in youth would be valuable. Such investigations should also include considerations into the developmental sensitivity of these associations. Finally, to better understand the implications of our findings for interventions it would be advantageous to study changes in both safety behaviour types over the course of psychological interventions for SAD and their relationship to treatment outcomes.

Our findings also give rise to a range of important clinical implications. Our findings suggest that youth with SAD are likely to use both ‘avoidance’ and ‘impression-management’ safety behaviours, with relatively greater use of ‘impression-management’ behaviours in older adolescents. The findings provide support for interventions that target both of these safety behaviours, such as cognitive therapy for SAD in adolescence (Leigh and Clark, 2016 Leigh et al., accepted). Such interventions may be informed by use of specific social anxiety safety behaviour questionnaire measures such as the SBQ. Findings that older adolescents showed greater relative use of ‘impression-management’ safety behaviours as well as higher scores on all SAD process and symptom measures highlights the need for developmentally tailored interventions and for early intervention. As ‘impression-management’ behaviours may be less noticeable to observers, it should also be considered that parent- or teacher-report of SAD symptoms may become less reliable in older adolescents. Use of impression management safety behaviours by an adolescent may mask the severity of their social anxiety, and they may ‘suffer in silence’. This perhaps points to the value of proactive screening initiatives to identify socially anxious adolescents in need of help, rather than relying on teachers or parents to detect behavioural signs. Findings of the negative impact on social relationships associated with ‘avoidance’ safety behaviours suggest that young people with SAD are likely to be experiencing victimisation, and so some liaison with schools to address this may be an important component of therapy.

## Figures and Tables

**Table 1:** Descriptive statistics and between-sample comparisons of questionnaire measures.

	Younger Community M (SD)	Older Community M (SD)	Clinical M (SD)	*P* value
**Social Anxiety Symptoms**	40.98 (28.74)^a^	52.64 (32.30)^b^	95.49 (25.78)^c^	<.001
**Social Anxiety-Related Cognitions**	29.84 (26.36)^a^	36.23 (26.85)^b^	54.94 (20.04)^c^	<.001
**Social Safety Behaviours**	1.01 (0.48)^a^	1.22 (0.49)^b^	1.49 (0.40)^c^	<.001
**General Anxiety**	18.13 (14.44)	21.69 (15.76)	-	<.01
**Depression**	7.15 (6.45)	10.06 (7.25)	-	<.001
**Friendship Quality**	26.65 (7.88)	26.31 (7.49)	-	.58
**Peer Victimisation**	4.62 (1.57)	4.44 (1.72)	-	<.05
**Social Satisfaction**	22.22 (4.22)	21.43 (5.09)	-	.19

*Note.^a,b,c^:* Different letters in superscript indicate significant differences (at least p <.05 after Bonferroni corrections) between values as indicated by the Kruskal-Wallis test. Social anxiety symptoms = LSAS-CA (Liebowitz Social Anxiety Scale for Children and Adolescents); Social anxiety-related cognitions = CASCQ (Child & Adolescent Social Cognitions Questionnaire – Belief ratings); Social safety behaviours = SBQ (Social Behaviour Questionnaire); General anxiety = SCARED (The Screen for Child Anxiety Related Disorders excluding social anxiety items); Depression = SMFQ (Short Moods and Feelings Questionnaire); Friendship quality = FQQ (Friendship Quality Questionnaire) – Validation & Caring Subscale; Peer victimisation = Peer Victimisation Scale; Social satisfaction = Social Satisfaction Scale.

**Table 2:** Item loadings onto ‘Avoidance’ and ‘impression-Management’ factors in EFA on first randomly-selected half of the Community Sample.

	Loading Magnitude	
Item	Avoidance	Impression-Management
Try not to attract attention	**.51**	.11
Make an effort to get your words right	.22	**.41**
Check that you are coming cross well	.01	**.54**
Avoid eye contact	**.51**	.05
Talk less	**.59**	.00
Avoid asking questions	**.64**	-.02
Try to picture how you appear to others*	.33	.37
Grip cups or glasses tightly	**.44**	.17
Position yourself so as not to be noticed	**.74**	.03
Try to control shaking*	.40^1^	.17
Choose clothes that will prevent or hide sweating	**.42**	.11
Wear clothes or makeup to hide blushing	**.56**	-.13
Rehearse sentences in your mind	.37	**.40**
Check what you are going to say	.29	**.49**
Blank out or switch off mentally	**.48**	.12
Avoid talking about yourself	**.58**	.02
Keep still*	.30	.22
Ask lots of questions*	-.35	.37
Stay on the edge of groups	**.60**	-.01
Avoid pauses in speech*	.21	.37
Hide your face	**.74**	-.05
Try to think about other things	**.45**	.20
Use alcohol/drugs to manage anxiety*	.20	.04
Talk more	-.30	**.45**
Try to fit in and ‘act normal’	.14	**.56**
Try to stay in control of your behaviour	.04	**.48**
Make an effort to come across well	-.08	**.74**
Planning things to talk about before a conversation	.20	**.49**

*Note.*

* Item did not load sufficiently onto either factor.

1 rounded up from .396 for the purpose of this table, but not included in Factor 1 as it did not meet the cut-off criteria of >.40.

**Table 3:** Item loadings onto the two factors following CFA in the Community Sample and Clinical Sample.

	Loading Magnitude			
	Community Sample		Clinical Sample	
**Avoidance Factor**				
Try not to attract attention	.64		.67	
Avoid eye contact	.63		.37	
Talk Less	.65		.80	
Avoid asking questions	.71		.63	
Grip cups or glasses tightly	.62		.54	
Position yourself so as not to be noticed	.80		.71	
Choose clothes that prevent / hide sweating	.57		.60	
Wear clothes or makeup to prevent or hide blushing	.59		.60	
Blank out or switch off mentally	.63		.50	
Avoid talking about yourself	.65		.55	
Stay on the edge of groups	.63		.47	
Hide your face	.74		.60	
Try to think about other things	.60		.16	
**Impression-Management Factor**				
Make an effort to get your words out right		.63		.74
Check that you are coming across well		.53		.45
Rehearse sentences in your mind		.87		.93
Check what you are going to say		.83		.88
Try to fit in and act normal		.62		.52
Try to stay in control of your behaviour		.52		.59
Make an effort to come across well		.61		.67
Planning things to talk about before a conversation		.72		.73

**Table 4:** Spearman’s Rho correlations between ‘avoidance’, ‘impression-management’ and all other questionnaire variables.

	COMMUNITY SAMPLE		CLINICAL SAMPLE	
	Avoidance	Impression-Management	Avoidance	Impression-Management
**Impression-Management**	.79***	-	.53***	-
**Social Anxiety Symptoms**	.75***	62***	.64***	41***
**Social Anxiety-Related Cognitions**	.65***	58***	.63***	54***
**Social Safety Behaviours**	.93***	90***	.81***	78***
**General Anxiety^1^**	.72***	.68***	40***	48***
**Depression^2^**	.65***	.56***	.37**	48***
**Friendship Quality**	-28***	-19***	-	-
**Peer Victimisation**	.24***	-19***	-	-
**Social Satisfaction**	-.42***	-.34***	-	-

*Note*.

* p<.05

** p<.01

*** p<.001. Social Anxiety Symptoms = LSAS-CA (Liebowitz Social Anxiety Scale for Children and Adolescents); Social anxiety-related cognitions = CASCQ (Adolescent Social Cognitions Questionnaire - Belief ratings); Social safety behaviours = SBQ (Social Behaviour Questionnaire); General anxiety = SCARED (The Screen for Child Anxiety Related Disorders) in Community Sample excluding social anxiety subscale items

1 RCADS-C (Revised Children’s Anxiety and Depression Scale) Anxiety subscale in Clinical Sample excluding social anxiety subscale items

2 Depression = SMFQ (Short Moods and Feelings Questionnaire) in Community Sample and, RCADS-C (Revised Children’s Anxiety and Depression Scale) Depression subscale in Clinical Sample; Friendship Quality = FQQ (Friendship Quality Questionnaire) - Validation & Caring Subscale); Peer victimisation = Peer Victimisation Scale; Social satisfaction = Social Satisfaction Scale.

**Table 5:** Multiple linear regressions of age group and social safety behaviour scores as predictors of ‘avoidance’ and ‘impression-management’.

	Estimate	B	SE	T value	P value
**Avoidance**					
**Constant**	-1.87	.00	.03	-65.64	< .001
**Social Safety Behaviours (Average)**	1.76	.93	.02	71.39	< .001
**Age Group (Younger vs. Older)**	< .01	< .01	.03	.32	.75
**Impression-Management**					
**Constant**	-1.76	.00	.03	-54.41	< .001
**Social Safety Behaviours (Average)**	1.67	.91	.03	59.78	< .001
**Age Group (Younger vs. Older)**	-.08	-.04	.03	-2.53	< .05
